# Reliability and Discriminative Ability of a New Method for Soccer Kicking Evaluation

**DOI:** 10.1371/journal.pone.0147998

**Published:** 2016-01-26

**Authors:** Ivan Radman, Barbara Wessner, Norbert Bachl, Lana Ruzic, Markus Hackl, Arnold Baca, Goran Markovic

**Affiliations:** 1 Department of Exercise Physiology, Institute of Sport Science, University of Vienna, Vienna, Austria; 2 Austrian Institute of Sports Medicine, Vienna, Austria; 3 Department of Exercise Physiology, School of Kinesiology, University of Zagreb, Zagreb, Croatia; 4 Department of Biomechanics, Institute of Sport Science, University of Vienna, Vienna, Austria; 5 Motor Control and Human Performance Laboratory, School of Kinesiology, University of Zagreb, Zagreb, Croatia; University of the Balearic Islands, SPAIN

## Abstract

The study aimed to evaluate the test–retest reliability of a newly developed 356 Soccer Shooting Test (356-SST), and the discriminative ability of this test with respect to the soccer players' proficiency level and leg dominance. Sixty-six male soccer players, divided into three groups based on their proficiency level (amateur, n = 24; novice semi-professional, n = 18; and experienced semi-professional players, n = 24), performed 10 kicks following a two-step run up. Forty-eight of them repeated the test on a separate day. The following shooting variables were derived: ball velocity (BV; measured via radar gun), shooting accuracy (SA; average distance from the ball-entry point to the goal centre), and shooting quality (SQ; shooting accuracy divided by the time elapsed from hitting the ball to the point of entry). No systematic bias was evident in the selected shooting variables (SA: 1.98±0.65 vs. 2.00±0.63 m; BV: 24.6±2.3 vs. 24.5±1.9 m s^-1^; SQ: 2.92±1.0 vs. 2.93±1.0 m s^-1^; all p>0.05). The intra-class correlation coefficients were high (ICC = 0.70–0.88), and the coefficients of variation were low (CV = 5.3–5.4%). Finally, all three 356-SST variables identify, with adequate sensitivity, differences in soccer shooting ability with respect to the players' proficiency and leg dominance. The results suggest that the 356-SST is a reliable and sensitive test of specific shooting ability in men’s soccer. Future studies should test the validity of these findings in a fatigued state, as well as in other populations.

## Introduction

The clear aim of a soccer match is to score more goals than the opposing team [1]. Consequently, soccer shooting (or kicking) ability, which includes both shooting speed and shooting accuracy, represents one of the most important soccer-specific movement qualities [2]. So far, a number of soccer-specific shooting tests have been introduced and scientifically evaluated (for review, see Ali 2011 [1], and Russell and Kingsley 2011 [3]). Notably, some authors focused solely on shooting speed [4,5], while others placed more attention on shooting accuracy in simple static situations [6,7,8,9] or on the complex interaction of various soccer skills (i.e., interaction of shooting accuracy with passing, ball control, and decision making) [10,11,12].

Of them, the most often cited is the Loughborough Soccer Shooting Test (LSST) [10]. The LSST assesses shooting skill on the basis of performance time as well as positive and negative points allocation for technical performance and errors. Although the reliability of LSST has been proven to be acceptable in male soccer players [10], several researchers have criticized its limited practical application due to the absence of information on absolute measures of technical proficiency, such as ball speed and accuracy [3,12]. The test also involves tasks of double ball rebounding, dribbling, and direction change techniques with shooting performance. In addition to its obvious advantages in overall shooting skill evaluation, these complex activities might interfere with kicking ability and likely cause noise if one aims to solely evaluate the shooting performance. The above mentioned complexity might reduce the LSST applicability while tracking changes elicited by the specific shooting training or fatigue/diet intervention. Russell et al. [12] additionally suggest the need for shooting performance tests that are expressed in ecologically valid units. This may (i) enable direct comparison of the performance among players of different proficiency levels and (ii) allow objective judgment on the effects of different training, competition or nutrition interventions.

To address the above-mentioned shortcomings of LSST, the authors proposed a test that uses comprehensive video analysis for measuring shooting accuracy, defined as the distance of the ball centre from the centre of the target positioned in the goal corner [12]. Although this shooting test indeed provides more interpretable outcome measures and might increase the comparativeness of the data, its practical usage is seriously limited by both the amount and price of the technical equipment needed. Equipment such as the two ball release systems, system for digitizing video footage, as well as several video cameras is sophisticated and expensive to acquire in the market. Hence, it is not accessible to a wide range of researchers or practitioners. Furthermore, the authors of the above-mentioned test incorporated four target lights in the corners of the goal. However, actual shots in the game do not necessarily require aiming the ball at one particular corner of the goal. Indeed, targeting in soccer depends on numerous factors including player’s own position, the position of teammates and opponents, ball’s position, goalkeeper’s attention, player’s self-confidence and overall readiness to take risk when shooting. Finally, applying the general definition of accuracy, i.e., ‘‘distance of the ball from the target”, in soccer seems generally arguable. The approach observed in two studies of Wood and Wilson [13,14] suggested that a higher accuracy-score should reflect shots that were placed further from the goalkeeper’s reach, where they would have a better chance of scoring.

This brief literature review highlights the limitations of the existing soccer shooting tests. Accordingly, it seems apparent that the future of soccer shooting evaluation should take into account the risks taken when shooting (i.e., during the match), ecological quantification of missed shots, distance of the ball from the goalkeeper’s reach, shooting speed, and standardized difficulty of goal-saving in terms of the necessary goalkeeper reaction. In addition, evaluation of soccer shooting ability should be based on simple, reliable, valid and sensitive field tests that can be applied in practice with a reasonable budget. Having in mind those methodological issues, we developed a new soccer shooting test (i.e., 356 Soccer Shooting Test; 356-SST). Within the present study, we evaluated the inter-session reliability and discriminative ability of the 356-SST on adult male soccer players.

## Materials and Methods

### Experimental approach to the problem

This study contained two separate sections: the assessment of (1) test-retest reliability and (2) discriminative ability. Three groups of soccer players participated in the study: amateur (AP; n = 24), novice semi-professional (NSP; n = 18) and experienced semi-professional (ESP; n = 24). Test-retest reliability study involved 48 players (24 mixed NSP and ESP, and 24 AP), which is in accordance with the number of participants in similar studies [10,12]. Discriminative ability of the test was evaluated using three groups of different players with regard to their playing level. In addition, the shooting performances of 48 soccer players (24 mixed NSP and ESP vs. 24 AP) while shooting with both the preferable and non-preferable leg were also compared.

The experimental procedure consisted of two main trials that included the 356-SST performance with both the preferable leg and non-preferable leg. Either one or, at most, two days separated the test and retest. Measurements were performed on a dry artificial grass soccer pitch at the Gerhard Hanappi Sport Centre in Vienna, Austria. The data were collected over the period of constantly stable and dry weather conditions (no wind; mean outdoor temperature: 22.4±1.7°C). Participants were invited to come to the testing pitch in groups of three. They were asked to refrain from exhausting physical activities during the 2 days before testing. To minimize the effects of circadian rhythms and other similar sources of variation, participants attended the retest within a time difference of not more than ±1 h of the first test. All participants were equipped with their standard soccer training clothing and footwear. A 20-min standardized warm-up consisting of running tasks, stretching exercises and ball technique preceded both main trials. To reduce the learning effect, players were given two free opportunities with each leg to habituate themselves with the testing protocol before recording sequences. Both main trials required participants to complete a total number of 10 shots with each leg distinctively, first with preferred leg and then, 3 minutes later, with non-preferred leg.

### Subjects

Altogether, 66 injury-free outfield soccer players (age: 21.9±4.5 years, height: 179.8±6.1 cm, body mass: 75.3±8.3 kg) participated in this study. The players were engaged from different league-levels of Austrian soccer and grouped on the basis of their proficiency and experience level: amateur (AP; no paid contract with the soccer club; n = 24), novice semi-professional (NSP; age<19 years; n = 18) and experienced semi-professional (ESP; ≥5 years of semi-professional experience; n = 24). The Ethics Committee of the University of Vienna approved the study. All participants were informed about the potential risks of the study and written informed consent was obtained before entry into the study, in accordance with the Declaration of Helsinki.

### The 356 Soccer Shooting Test (356-SST)

**Fig 1:**
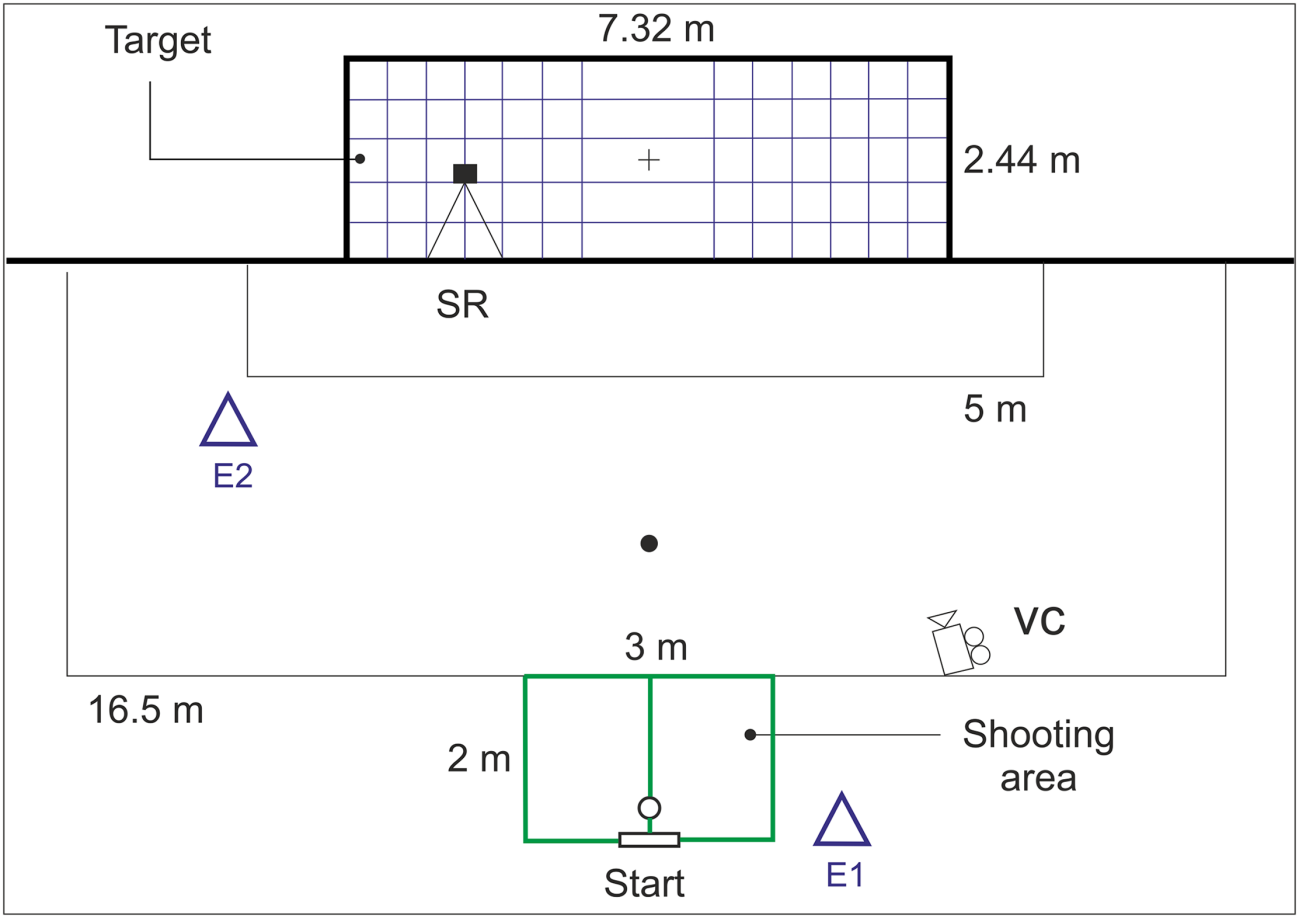
Layout of the newly developed 356-SST. Note: SR = sports radar gun; E1/E2 = examiner 1/examiner 2; VC = video camera; cross sign (+) = goal midpoint; Fig depicts the setting for shooting with the right leg.

The 356-SST aimed to measure three soccer-specific shooting variables: (1) *shooting accuracy* (SA), (2) *ball velocity* (BV) and (3) *shooting quality* (SQ). SQ was determined to be the most important variable and the main outcome measure of the test performance. Fig 1 illustrates the layout of the test. A marked rectangular shooting zone (2 × 3 m) was placed in the middle of the 16.5 m line. The overall test procedure contained 10 kicks, performed following two steps, with the elected leg from within the one-side shooting zone aiming towards the shooting target. The 356-SST required players to use two steps and one contact with the ball prior to the kick. At the beginning position, a player controls the ball under the foot elected for kicking. The first contact was used to adjust (roll forward) the ball in optimal position for the final interaction. The participant should hit the farther side of a standard soccer goal (7.33 x 2.44 m). Specifically, if the player uses right side shooting zone (i.e. right leg), he was instructed to aim the opposite (left) goal side, and vice versa (Fig 1). As the goalkeepers commonly train to cover goal side that is closer to the shooting position [10], particular shooting zone side was used as a reference to the goalkeeper’s position in the soccer game. Thus, the goal side that is opposite of the shooting zone side simulates an uncovered part of the goal. In order to enable recognition of scoring results, the goal plane was covered by an 8 mm thick, white net with dimensions corresponding to the goal size (Fig 1). Plastic cable-binders were used to fix the ending sides of the net on the goal frame. Both the left and right side of the net contained 30 fields, i.e. goal-scoring zones measuring 48.8 x 48.8 cm, distinguished with regard to both shooting and saving demands. In general, proximal scoring zones are easier to hit but offer to the goalkeeper better chance for saving, while distal zones are risky to miss and more demanding to hit but give the goalkeeper less chance to react successfully.

**Fig 2:**
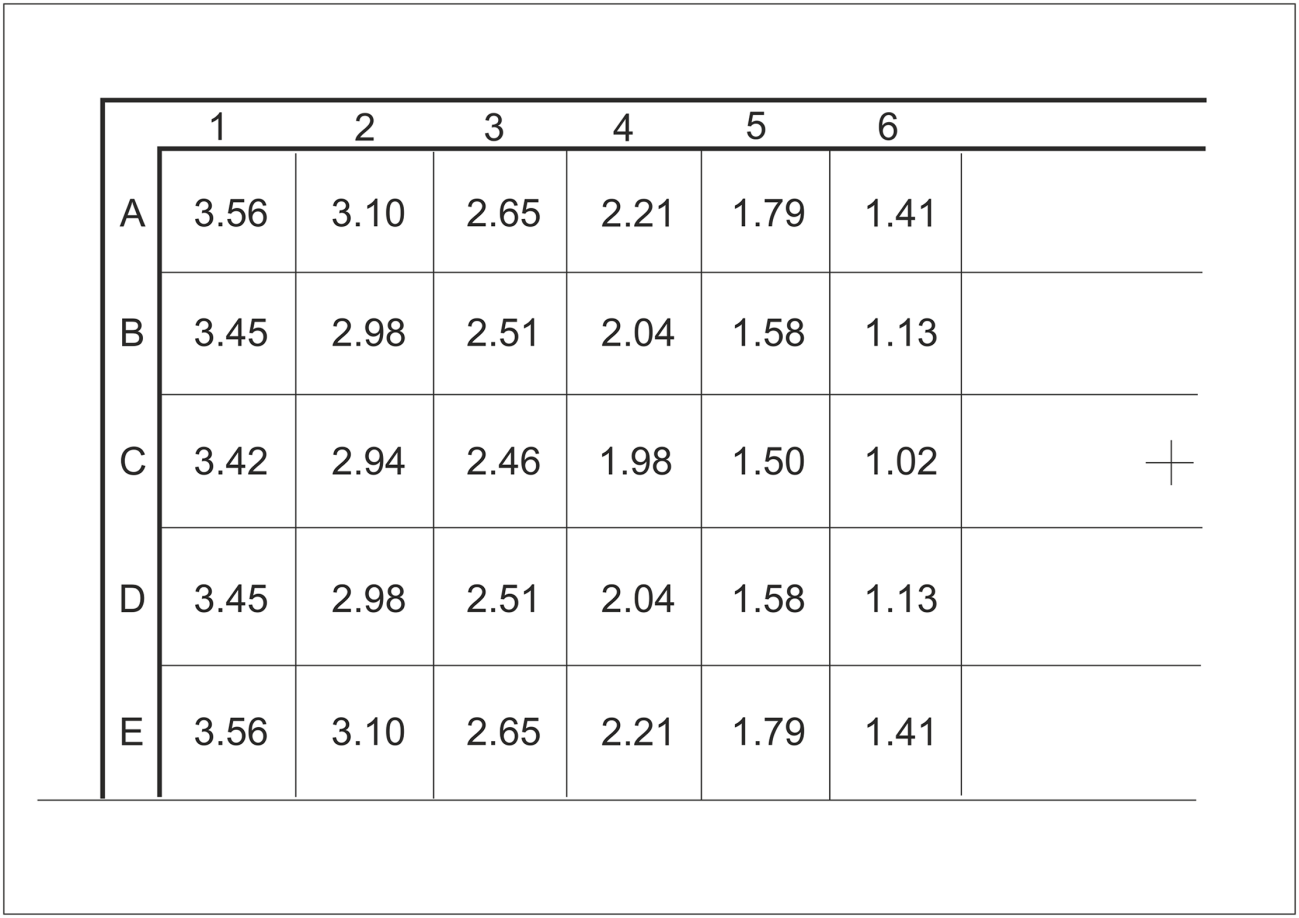
Scoring zones and determined distances (m) from the centre of each particular scoring zone to the goal-centre (cross sign).

Distances between the goal centre and the centre of each scoring zone were a priori calculated mathematically in two-dimensional space (see Fig 2). The specific aim while performing the test was to shoot the ball as sharply as possible, all the while maintaining control over the kick, into the most distant scoring-zone away from the goal centre. For each shooting attempt, the metre-distance was recorded according to the previously mentioned calculation. With the intention to encourage players to take risks similar to when shooting during official game play, only the best seven (according to the recorded distance) out of ten shooting attempts were used to calculate the final scores. The selected number of attempts is based on our pilot work and prior studies [12,15] that suggested mean success rate when shooting around 70%. To improve the ecological validity of the test and make the outcome measures easier to interpret, scores were expressed in metres (SA) and metres per second (SQ). Therefore, accuracy of soccer shooting technique was expressed as a mean distance of the centres of the 7 farthermost ball-hit scoring zones from the centre of the goal. Because the scoring zones that lay at the goal corners are the most distant fields for shooting and have already been identified as the most favourable placements for beating goalkeepers when shooting [10,12], their distance to the goal centre (3.56 m) was the theoretically maximal SA score of the 356-SST. To identify the ball-hit net fields, and by extension scoring zones, a video camera (Sony^™^, HDR-PJ260, Japan) was placed near the shooting zone (VC in Fig 1). With the purpose of BV assessment, a Radar Gun (Stalker^™^, Applied Concept, USA) was attached to a 1.1 m high stand and positioned behind the goal as depicted in Fig 1.

**Fig 3:**
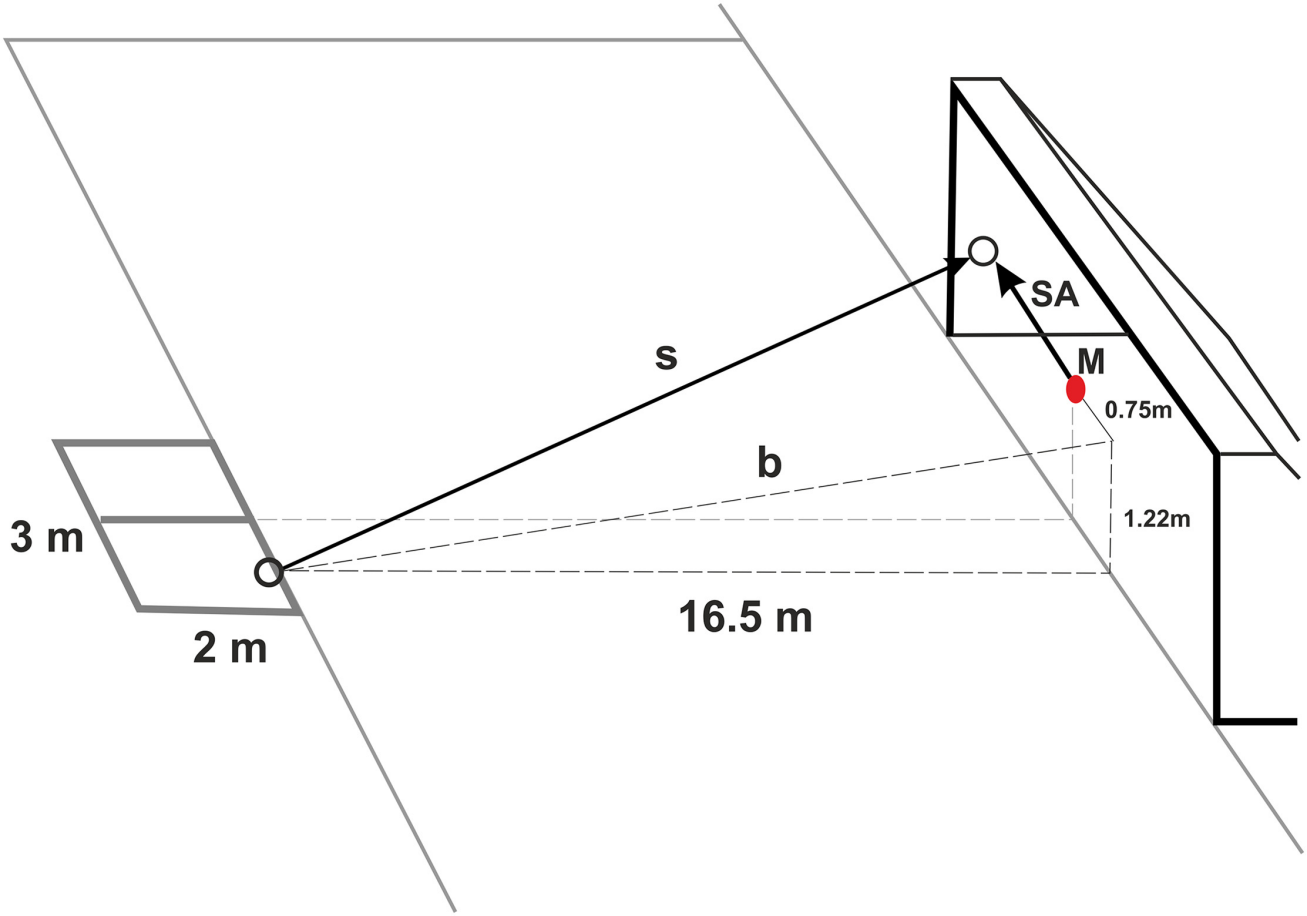
Geometrical calculation of theoretical ball route (s) following the foot-contact in the direction of the transversal line of the goal. Note: M (middle)—goal centre; s (spartium)^1^—conditional (theoretical) trajectory of the ball [m]; SA (shooting accuracy)—average distance from the ball–entry point to the goal centre [m]; b (bevel)^2^—the bevel under which the ball rises to the transversal goal line [m]; ^1^ b = √16.5^2^ + 1.22^2^; b = √272.25 + 1.4884; b = 16.55 m; ^2^ s = √ (SA + 0.75)^2^ + b^2^; s = √ (SA + 0.75)^2^ + 273.74

According to spatial parameters defined by the test setting, measured BV (m s^-1^) and recorded SA score (m), the shooting quality (SQ) score was calculated as the ratio of SA (mean distance of ball-hit scoring zones from the goal-centre) and the time (*t*) that elapsed between the moment the was hit and the point of entry (see Fig 3); **SQ = SA ⁄ t**, where *t* was calculated as a ratio of the conditional (theoretical) trajectory of the ball (*s*) and the recorded ball velocity (*v*). Thus, SQ is described as the mean speed of the theoretical goal mid-point needed to forestall the kicked ball before entering through the goal plane. Accordingly, the SQ outcome was expressed in metres per second.

As suggested previously [10,16], the selectivity criteria of shooting attempts included a minimal ball velocity of 64 km h^-1^ and shooting performance from a marked area. The time between each shot was 6 s, requiring players to complete the shot in 3 s and reset himself in ready position before the next examiner’s call. In cases where either the individual shot-performance did not meet one of the listed criteria or the ball missed the required scoring space, this particular attempt was scored as 0 m, as already proposed by Wood and Wilson [13,14]. The testing procedure required the involvement of two examiners (E1 and E2): E1 controlled the performance time, set the ball in basic position, and gave the start sign every 6 s, while E2 led the testing procedure (instructions, warm-up, video recording and ball collecting). To conduct the trial, 11 soccer balls (Adidas^™^, UEFA CLF 12, Adidas Group, Germany) and 4 plastic cones were used.

### Statistical analyses

The obtained data were processed using SPSS for Windows software (ver. 20.0, Chicago, Illinois, USA). In line with recommendations [17,18], the following three important components of reliability were calculated: (a) systematic bias, (b) within-individual variation (i.e. ‘‘absolute” measure of random error), and (c) retest correlation (i.e. ‘‘relative” measure of random error). Systematic bias was assessed using a paired samples t-test. Within-individual variation was assessed by a standard error of measurement (SEM) and coefficients of variation (CV). The SEM and CV were derived by two-way ANOVA. In particular, the participants represented a random effect, the number of tests in sequence was a fixed effect, and the test result, either raw (for the SEM) or log-transformed (for the CV), was the dependent variable (see Hopkins 2000 [18]). The SEM represented the square root of the mean square error term (RMSE) in the ANOVA output. The mean CV was calculated from the RMSE using the following formula: CV = 100×(e^RMSE^-1) ≈ 100×RMSE [18]. Finally, retest correlation was assessed using a two-way random model of the intraclass correlation coefficient (ICC_2,3_) described by Shrout and Fleiss [19]. Differences in shooting performance characteristics among the three qualitative groups as well as between mean scores obtained with preferred and non-preferred leg were compared by means of one-way ANOVA with a Bonferroni post-hoc analysis and paired samples t-test, respectively. The results were reported as means and standard deviations and the statistical significance was set at p<0.05.

## Results

Components of reliability of the 356-SST are presented in Table 1. No differences in participants’ performance outcomes (i.e., no systematic bias) between test 1 and test 2 were detected in terms of mean shooting quality (SQ; p = 0.94), mean shooting accuracy (SA; p = 0.72), and mean ball velocity (BV; p = 0.67). Within-individual variation (expressed as CV) for the three shooting performance measures ranged between 5.3% and 15.4% (Table 1). Finally, note that all three shooting performance measures had high to very high inter-session ICCs (Table 1).

**Table 1:** Reliability parameters of the 356-SST (n = 48).

Variable	Trial 1 mean ± SD	Trial 2 mean ± SD	Changes in means (95% CI)	SEM (95% CI)	ICC (95% CI)	CV (95% CI)
Shooting accuracy (m)	1.98 ± 0.65	2.00 ± 0.63	1.4% (-4.4–7.5%)	0.27 (0.23–0.34)	0.84 (0.73–0.91)	15.4% (12.6–19.6%)
Ball velocity (m/s)	24.6 ± 2.3	24.5 ± 1.9	-0.3% (-2.4–1.8%)	1.2 (1.0–1.5)	0.70 (0.69–0.82)	5.3% (4.4–6.6%)
Shooting quality (m/s)	2.92 ± 1.0	2.93 ± 1.0	1% (-4.2–6.7%)	0.37 (0.31–0.46)	0.88 (0.80–0.93)	14% (11.5–17.9%)

Note: SD = Standard deviation; ICC = Intra-class correlation coefficient; CV = Coefficient of variation; 95% CI = 95% confidence interval.

Shooting performance scores of the three groups of soccer players with various playing levels and experiences are displayed in Table 2. Note an increase in the main shooting performance variables with the increase in soccer proficiency level. The tested groups differed considerably from each other in SA, SQ, and BV (all p<0.01). *Post-hoc* analyses revealed significant differences in SA and SQ among all three qualitative groups, while a significant difference in BV was observed only between the amateur players and novice semi-professional players.

**Table 2:** Performance outcomes of the 356-SST with respect to the group proficiency level.

Group level	n	Age (y)	Shooting accuracy (m)	Ball velocity (m/s)	Shooting quality (m/s)
Amateur	24	23.8 ± 4.3	1.52 ± 0.42*	24.0 ± 2.1^#^	2.20 ± 0.69*
Novice semi-professional	18	18.2 ± 1.3	2.06 ± 0.47*	26.2 ± 1.5^§^	3.22 ± 0.78*
Experienced semi-professional	24	22.8 ± 4.8	2.46 ± 0.44*	25.1 ± 1.9	3.65 ± 0.62*
All	66	21.9 ± 4.5	2.00 ± 0.61	24.8 ± 2.1	2.98 ± 0.95

Note: Data are presented as the mean ± standard deviation.

* Significantly different from other two groups (p<0.05);

^#^ Significantly different from the group of novice semi-professional players (p<0.05);

^§^ Significantly different from the group of amateur players (p<0.05).

**Fig 4:**
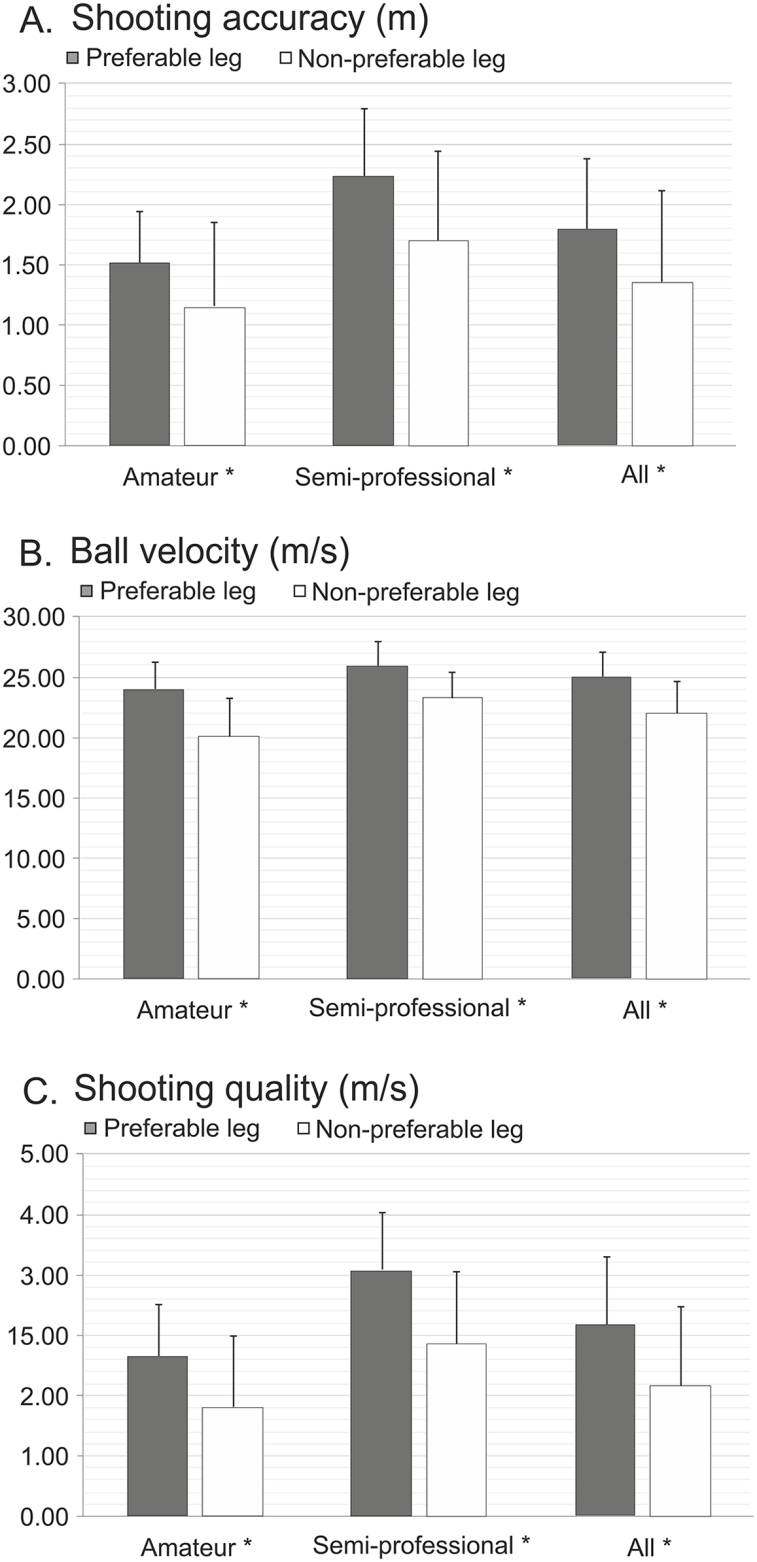
Performance outcomes of the 356-SST with the preferred vs. non-preferred leg presented as the mean ± standard deviation. Note: * Significant difference at p<0.05.

Likewise, shooting performance was significantly better when shooting bouts were taken using preferred rather than non-preferred within the same qualitative group (p<0.01) and within the overall sample of tested players (p<0.01), respectively (Fig 4).

## Discussion

The main finding of the present study was that the newly developed 356-SST may reliably be used to assess SA, BV and SQ in male, senior soccer players. Furthermore, our results indicate that soccer players of higher *vs*. lower proficiency, and with preferred *vs*. non-preferred leg, achieved a better score in tested soccer kicking variables, thereby supporting the discriminative ability of the 356-SST. These findings, together with their practical applications and limitations, are discussed in the following paragraphs.

### Reliability of the 356-SST

We have evaluated 3 components of test-retest reliability of the 356-SST: systematic bias, retest correlation, and within-individual variation [18]. Regarding systematic bias, we found no significant change in any of the three soccer-kicking variables between the test and retest. Observed relative change in means of kicking variables from test 1 to test 2 ranged from 0.3% for BV, over 1% for SQ to 1.4% for SA. Based on the data reported by Ali et al. [10] for LSST, changes in means between trials was ~9.5% (points per shot) and 0.8% (shot speed) for a combined group of elite and non-elite players. Similarly, the mean biases reported by Russell et al. [12] for an entire sample of professional and recreational players amounted to 8.7% for shooting success and 3% for shooting precision variables, while most outcome measures (dribbling, passing, shooting) produced biases less than 5%. In comparison with these results, the magnitude of all identified changes between two 356-SST tests is quite small (<1.5%) and may, from the practical point of view, be neglected. A similar change in means between repeated trials was reported only by researchers that employed simple soccer shooting tests from the stationary position [4,5].

The second component of reliability, i.e., retest correlation, was calculated using ICC. The relative reliability for all three soccer kicking measures was moderate to high (ICC = 0.70–0.88), considerably higher than those reported for previously developed tests that incorporated some measures of shooting accuracy (ICC = 0.26–0.38 [10,12]) and comparable, again, to those reported for ball velocity in stationary shooting tests (ICC = 0.87–0.95 [4,5]). The relatively low retest correlations of soccer shooting tests reported by Ali et al. [10] and Russell et al. [12] may be explained by the fact that they included additional task components prior to the kicking performance. Time- and energy-consuming tasks, such as running with direction change and double ball rebounding [10] or reacting to a sudden visual registration and then hitting the ultimate, risky target without prior ball-control [12], might have contributed to a relatively low relative reliability of these tests. In contrast, the newly developed 356-SST isolated only the essential components needed to assess the kicking skill without compromising the relative reliability of the test. Because it incorporated self-preferred ball adjusting, controlling the leg-ball distance while performing decisive steps, self-elected risk taking, aiming and kicking toward the empty side of the goal, the 356-SST seems valid and legitimate for reproducing the original shot performed during the game.

We also calculated the third component of reliability, i.e., within-individual variation, using both the SEM and CV. The calculated inter-session CVs ranged between 5.3% and 15.4%, being highest in SA. Regarding the ball velocity (BV), the observed CV is similar to (2.6–7.5% [4,5]) or lower (9.5% [12]) than those reported in previous research. Regarding SA, the observed CV is considerably lower than those reported by other studies for existing soccer shooting tests (57.8% by Ali et al. [10] and 23.5% by Russell et al. [12]). The observed within-individual variations for SA, BV, and SQ may also be considered acceptable in terms of the tracking of meaningful changes in soccer kicking ability. Several authors suggested that the thresholds for the smallest worthwhile effect on BV and SA are ~5% and ~20% [4,5], i.e., similar to or lower than the within-individual variations observed in the present study. Thus, all three measures of soccer shooting ability have good ability to detect real and meaningful differences or changes with feasible sample sizes. Based on the above-discussed results, we may conclude that the 356-SST represents a reliable test for the evaluation of soccer kicking performance in male soccer players.

### Discriminative ability of a 356-SST

The second important finding of this study is that all 356-SST variables identify, with adequate sensitivity, differences in soccer shooting ability with respect to players' proficiency level and leg dominance. Specifically, compared to amateur and novice semi-professional players, the shooting series made by more experienced semi-professional players were performed with 61.8% and 19.4% greater accuracy and would practically require 65.9% and 13.4% faster goalkeeper’s reactions, respectively. Furthermore, novice semi-professional players performed shots with 35.5% greater accuracy and 8.8% higher velocity than amateur players, giving them nearly a 46.4% better chance to score. Note that previous studies mainly failed to reveal differences in kicking performance among players of different proficiency levels [10,20–22]. Possible reasons for their relatively low discriminative ability could be lower reliability of soccer kicking tests and (in some cases) players’ maturation level. Recently, Berjan et al. [4] evaluated a composite test of kicking performance in a large sample of young players and showed good discriminative ability of both SA and BV variables with respect to age.

The selected 356-SST variables also showed good sensitivity with respect to leg dominance, regardless of players’ proficiency level. On average, players achieved 32.6%, 13.9% and 46.6% better scores in SA, BV, and SQ with the preferred vs. non-preferred leg, respectively. This is despite the fact that soccer coaches strive to develop symmetry in players’ shooting ability with both the preferred and non-preferred leg. Berjan et al. [4] also recently reported leg asymmetries in SA and BV during instep kick in young soccer players. Overall, similar to the reliability data, the discriminative ability analysis supports a routine use for all evaluated variables of the 356-SST.

### Limitations and practical applications

It should be noted that the newly developed 356-SST belongs to the category of shooting ability tests (i.e., it measures shooting velocity and accuracy during instep kicking from the stationary position), rather than to the category of complex shooting skill tests [1,10,12,23]. From that perspective, one could argue that the 356-SST has lower ecological validity compared with the latter category of shooting tests. On the other hand, we should also keep in mind that the existing complex shooting skill tests have methodological and practical limitations that prevent their application in everyday soccer practice (see Introduction for details). Thus, it seems that both types of tests may contribute to the comprehensive evaluation of shooting performance in the practice of soccer. However, because it precisely contextualizes test outcomes, and requires a reasonable budget, the 356-SST might have practical advantages concerning ecological validity and feasibility. In particular, the 356-SST has introduced an original variable (i.e. SQ) that combines different time and spatial parameters to express the quality of shooting performance. A novel measure, which pertains to the ‘‘speed of the goalkeeper reaction needed to prevent goal scoring”, seems quite understandable and easy to interpret from the practical point of view. It enabled users to observe only one, well-explanatory variable instead of analysing the multiple measures needed to describe shooting success. Lastly, the reliability of 356-SST has been confirmed on men in a non-fatigued state, mostly because of closer comparison with the existing literature. Further studies are needed to evaluate this test both in the fatigued state and on other soccer populations (i.e., youth and females), as well as to determine its sensitivity to typical diet and training interventions.

## Conclusions

In conclusion, the newly developed 356-SST proved to be a reliable method for the evaluation of soccer shooting ability in male soccer players. Additionally, the test proved to be sufficiently sensitive to detecting differences in soccer shooting ability with respect to players' proficiency level and leg dominance. As a result, the 356-SST has a good potential to be applied in both research and practical settings for the purpose of selecting and profiling soccer players, as well as for examining the effects of various training or diet interventions aimed at enhancing soccer performance. Of particular practical importance for coaches could be the fact that the test actually measures the speed of the goalkeeper motor reaction needed to prevent goal scoring. Logically, the faster the goalkeeper reaction needed, the better the performance. This measure enables soccer coaches and players to interpret testing outcomes authentically.
